# Supplementary material for: C. elegans germ granules sculpt both germline and somatic RNAome
Source: Nat Commun. 2023 Sep 25;14:5965. doi: 10.1038/s41467-023-41556-4 (PMC10520050; doi:10.1038/s41467-023-41556-4)
Supplement: Supplementary file 1 — Supplementary information [file 41467_2023_41556_MOESM1_ESM.pdf]

Supplementary Information for:

C. elegans germ granules sculpt both germline and somatic RNAome

Ian F. Price[1,2,3#], Jillian A. Wagner[1,2#], Benjamin Pastore[1,2,3], Hannah L. Hertz[1,2], Wen Tang[1,2,4*]

[1]Department of Biological Chemistry and Pharmacology, Columbus, OH 43210, USA.
[2]Center for RNA Biology, The Ohio State University, Columbus, OH 43210, USA.
[3]Ohio State Biochemistry Program, Columbus, OH 43210, USA.
[4]Corresponding author.
[#]Equal contribution.

Note that the citations refer to the references in the main manuscript

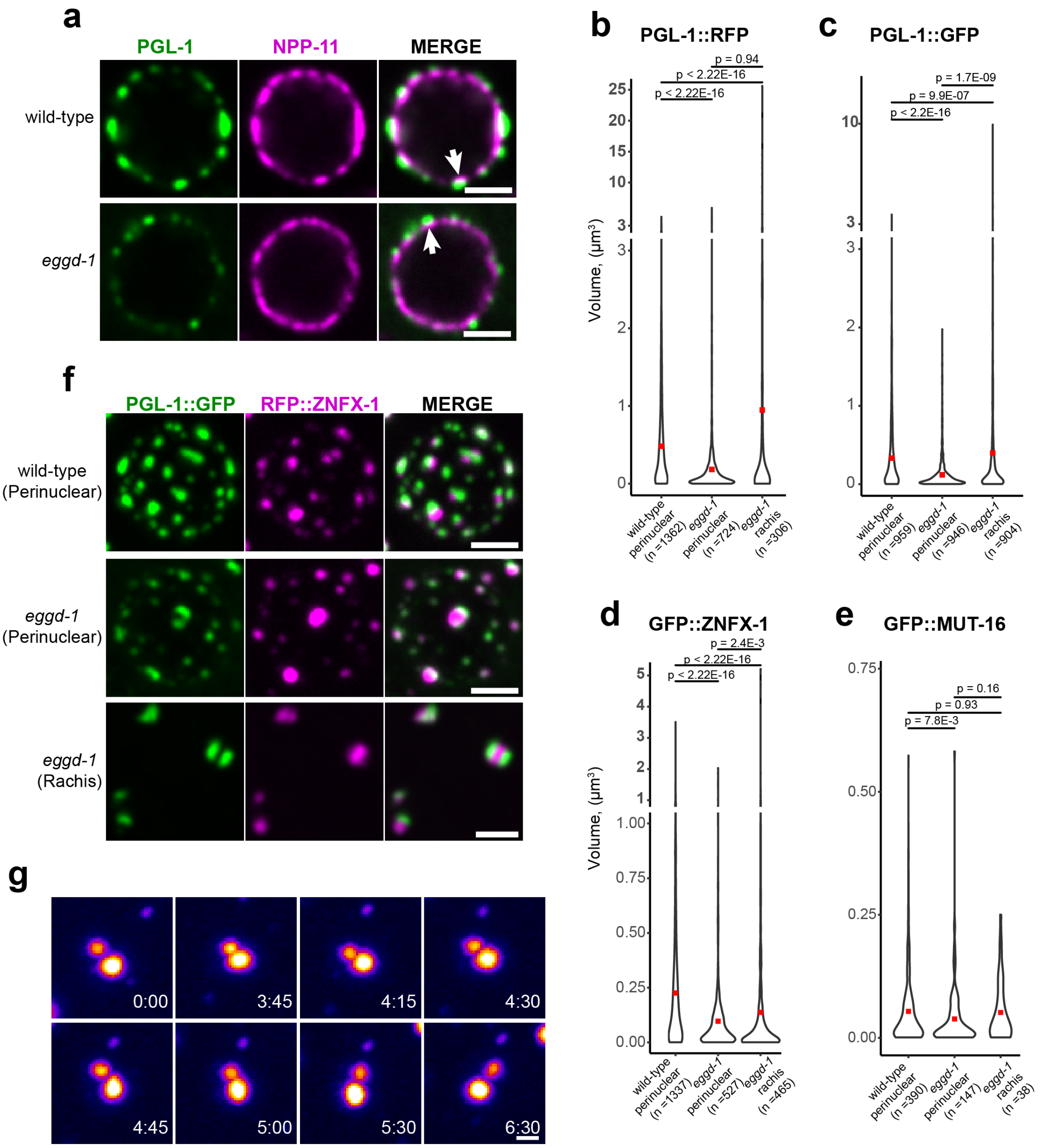

Supplementary Fig. 1: The volume and dynamics of germ granules.

a. Single confocal slice of a pachytene germ cell nucleus from wild-type or *eggd-1* mutant worms expressing NPP-11::wrmScarlet and PGL-1::GFP. Arrows indicate select areas with surface-to-surface contact between NPP-11 and PGL-1, which can be observed in wild-type and *eggd-1* mutants. Images are representative of 6 worms imaged over one experiment. Scale bar = 2 µm.

b-e. Violin plots showing the volume of PGL-1::RFP (**b**), PGL-1::GFP (**c**), GFP::ZNFX-1 (**d**), and GFP::MUT-16 (**e**) granules in wild-type and *eggd-1* mutants germ lines. The volume of granules with perinuclear or rachis localization in *eggd-1* mutants are

quantified and plotted separately. The red square indicates the mean volume of granules. The Wilcoxon signed-rank test was used to calculate p-values. n = number of granules. Source data are provided as a Source Data file.

f. Maximum intensity projections of confocal fluorescent micrographs showing PGL-1::GFP and RFP::ZNFX-1 in wild-type or *eggd-1* mutant germ lines. Images depict single nuclei (top two) or granules of PGL-1::GFP and RFP::ZNFX-1 at the *eggd-1* rachis (bottom). Scale bar = 2 μm. n = 5 worms imaged over two independent experiments.

g. Time-lapse confocal imaging of PGL-1::RFP granules localized to the *eggd-1* rachis. Over the time course, two granules collided, but did not coalesce. Time is in minutes : seconds. Scale bar = 1 μm. Images are representative of observations in two independent timelapse experiments.

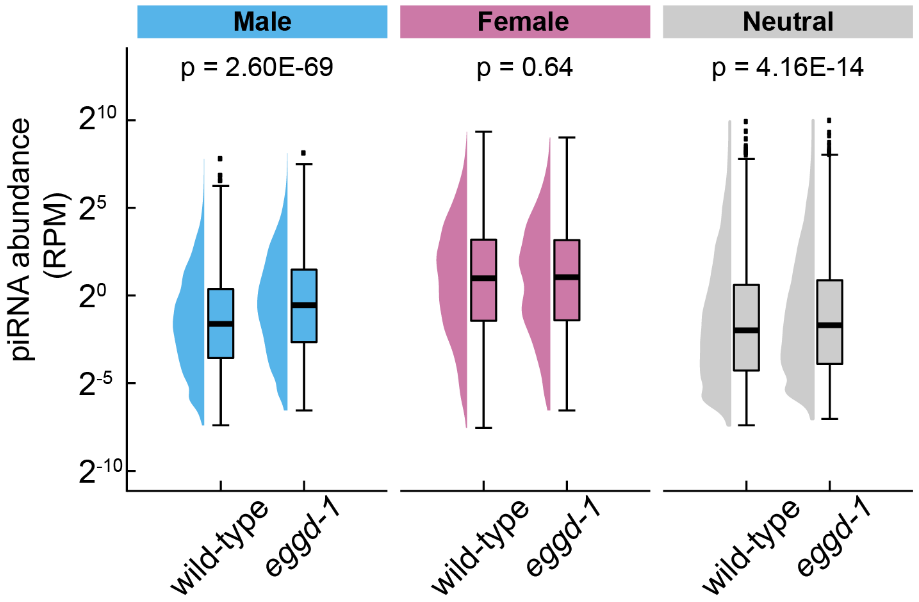

Supplementary Fig. 2: Expression of spermatogenic specific, oogenic specific gender neutral piRNAs.

Boxplots showing the abundance of spermatogenic specific (n = 3,594) oogenic specific (n = 1,682) or gender neutral (n = 7,583) piRNAs in wild-type and *eggd-1* mutants[53]. The median piRNA abundance is shown as a solid black line within each box. Each box displays the interquartile range of the data (between the 25th and 75th percentile). A two-tailed t-test was used to derive p-values.

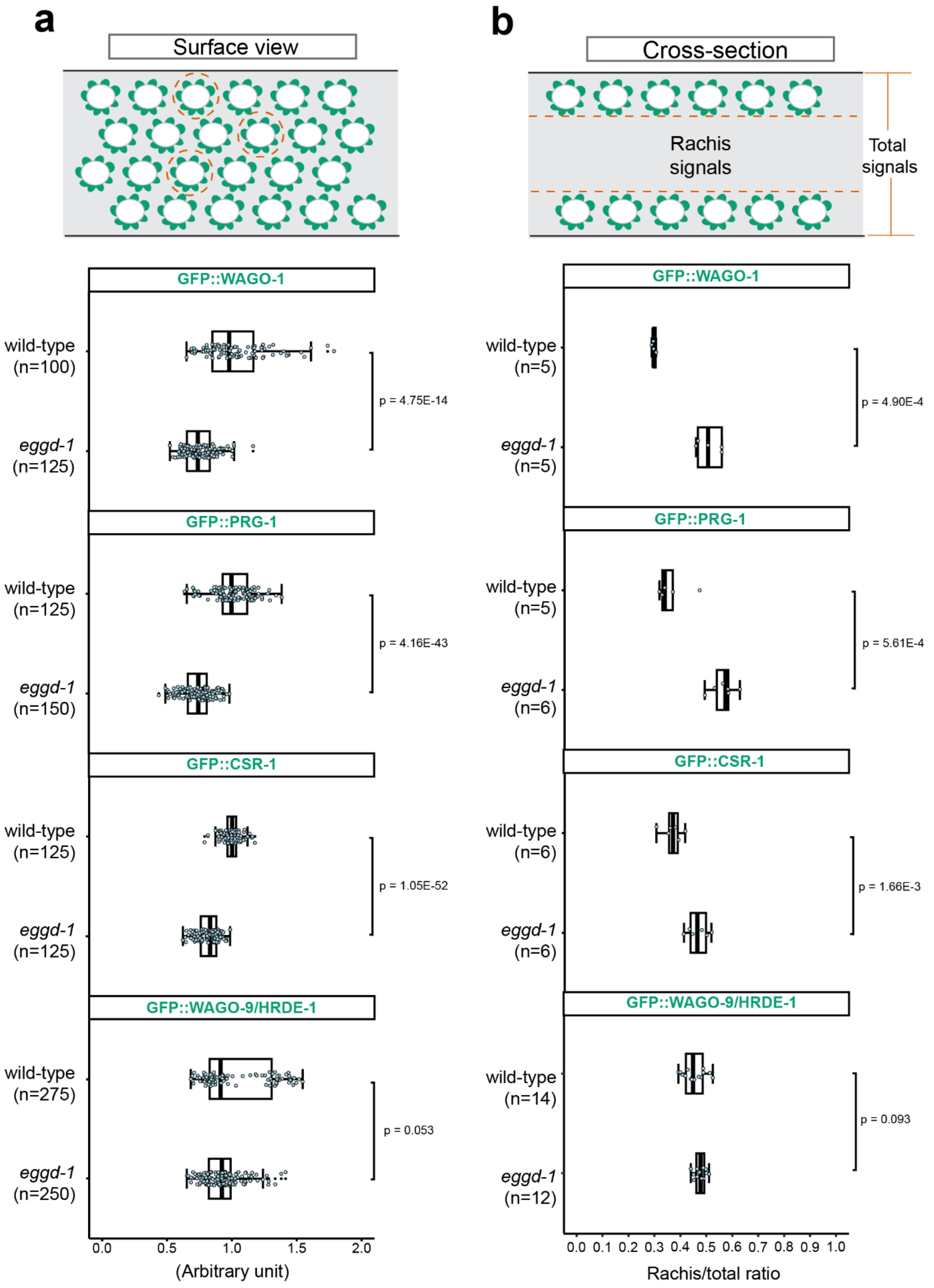

Supplementary Fig. 3: Quantification of Argonaute expression in wild-type and *eggd-1* mutants.

a. Schematic depicting method used to quantify the perinuclear or nuclear intensity of GFP tagged proteins (top panel). Individual germline nuclei were selected and the integrated density was plotted by box and whisker plots, which show the intensity of GFP::WAGO-1, GFP::PRG-1, GFP::CSR-1, and GFP::WAGO-9/HRDE-1 in wild-type and *eggd-1* adult hermaphrodites. A two-tailed t-test was used to determine p-values. Source data are provided as a Source Data file.

b. Schematic depicting method used to quantify rachis signals over the total signals of GFP::WAGO-1, GFP::PRG-1, GFP::CSR-1, and GFP::WAGO-9/HRDE-1 in wild-type and *eggd-1* adult hermaphrodites. For each cross-section, the gonad and rachis were outlined and integrated density was measured (top panel). The rachis/total ratio was plotted in a box and whisker plot (bottom). A two-tailed t-test was used to determine p-values. Source data are provided as a Source Data file.

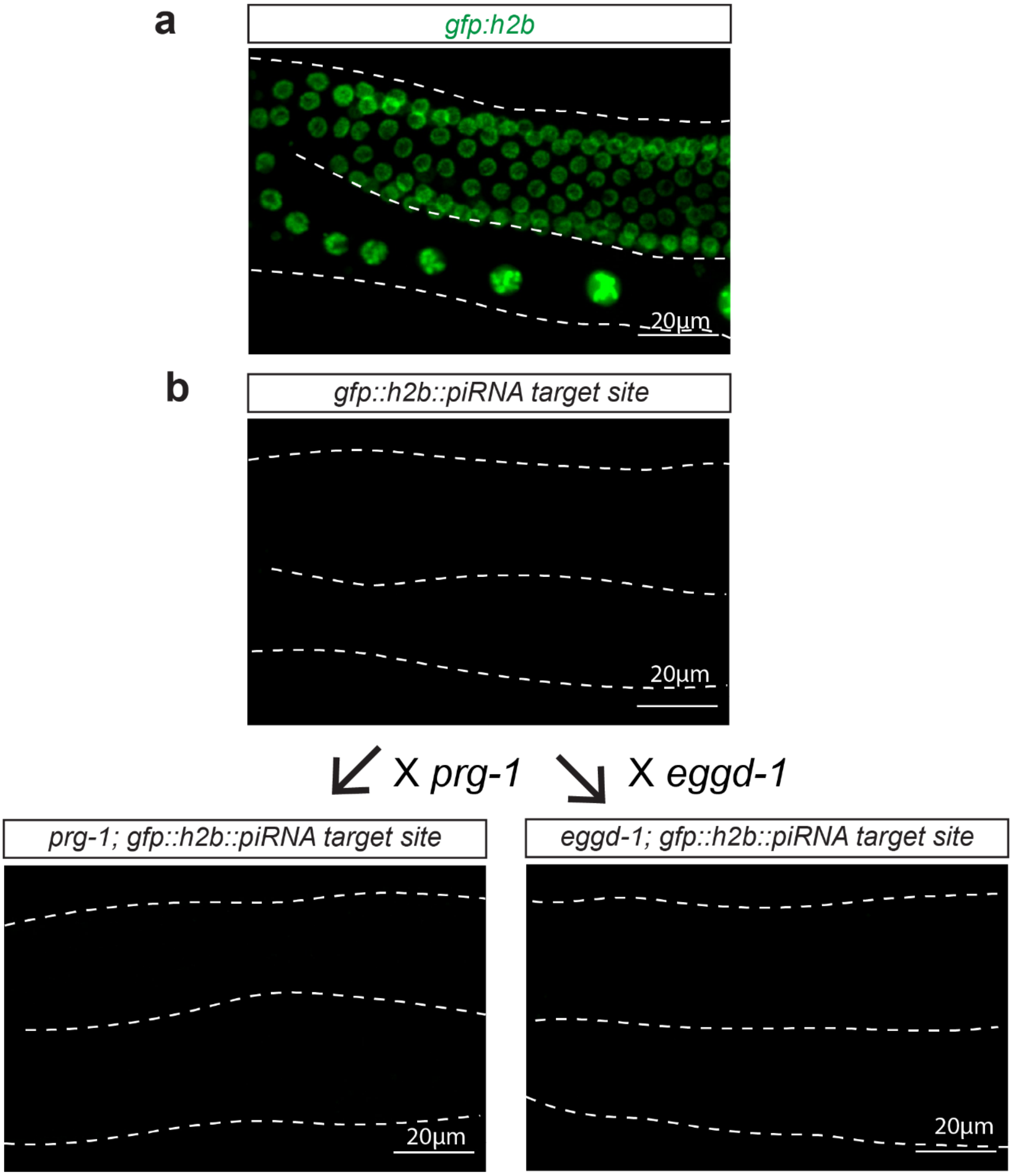

c

Strain	% GFP ON	n
gfp:h2b-58	100%	9
gfp::h2b::piRNA target site	0%	7
prg-1; gfp::h2b::piRNA target site	0%	8
eggd-1; gfp::h2b::piRNA target site	0%	7

Supplementary Fig. 4: The role of *eggd-1* in PRG-1/piRNA-mediated silencing.

a. Maximum intensity projection of *gfp::h2b* transgenic strains. White dashed lines outline gonad. Image is representative of 10 worms for each n independent experiment under conditions where GFP was determined to be 100% ON (refer to panel (**c**)). Scale bar = 20µm.

b. Maximum intensity projection of *gfp::h2b::piRNA target site* transgenic strains. Silencing of the *gfp::h2b::piRNA target site* transgene was initiated in wild-type and crossed to *prg-1* or *eggd-1* mutants. Image is representative of 10 worms for each n independent experiment under conditions where GFP was determined to be 0% ON (refer to panel (**c**)). White dashed lines outline gonads. Scale bar = 20µm.

c. The table indicates the percentage of worms with GFP ON for each strain over n independent experiments.

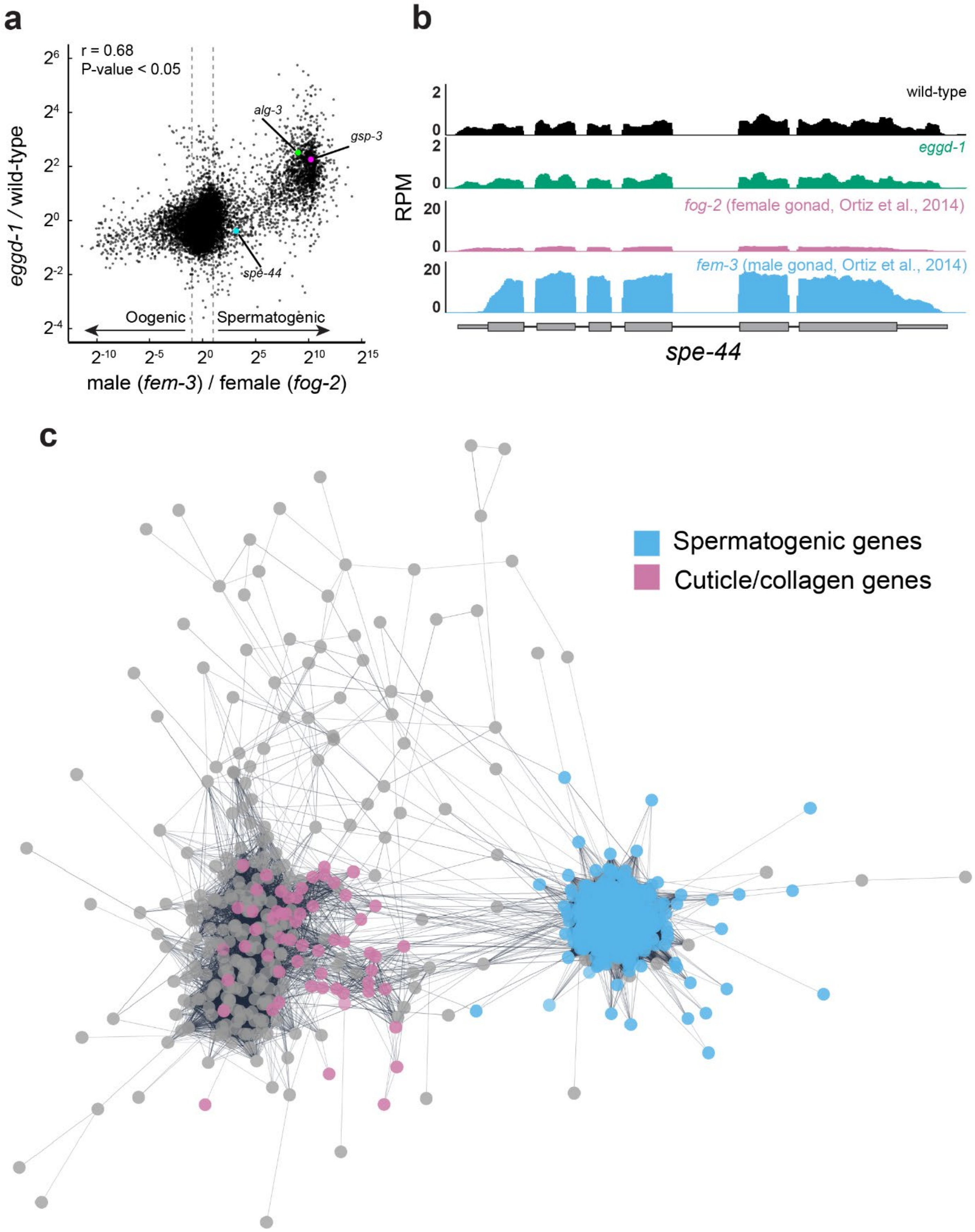

Supplementary Fig. 5: Upregulation of spermatogenic genes in *eggd-1* mutants.

a. Scatterplot showing the correlation between the fold-change of transcripts in *eggd-1* relative to wild-type and the fold-change of transcripts in male (*fem-3*) compared to female (*fog-2*). Dashed lines are located at the cutoff $\log_2$(*fem-3*/*fog-2*) ≥ 1 and $\log_2$(*fem-3*/*fog-2*) ≤ -1 which are used to define spermatogenic or oogenic transcripts by Ortiz et al.[76]. Green, magenta, and cyan dots indicate *alg-3, gsp-3,* and *spe-44* transcripts, used as specific examples in Fig. 5d, 5e, and Supplementary Fig. 5b, respectively. *r* indicates Pearson correlation coefficient.

b. Browser track showing the abundance of *spe-44* transcript as measured by RNA sequencing from wild-type and *eggd-1* mutant animals as well as *fog-2* and *fem-3* gonads[76]. The coverage is the average of 2 sequencing replicates (*wild-type* and *eggd-1*) or 7 sequencing replicates (*fog-2* and *fem-3* mutants).

c. STRING protein-protein interaction network analysis of mRNAs upregulated in *eggd-1* mutants. Spermatogenic genes defined by Ortiz et al., are labeled in cyan[76]. Genes encoding collagen or structural cuticle constituents are labeled in magenta.

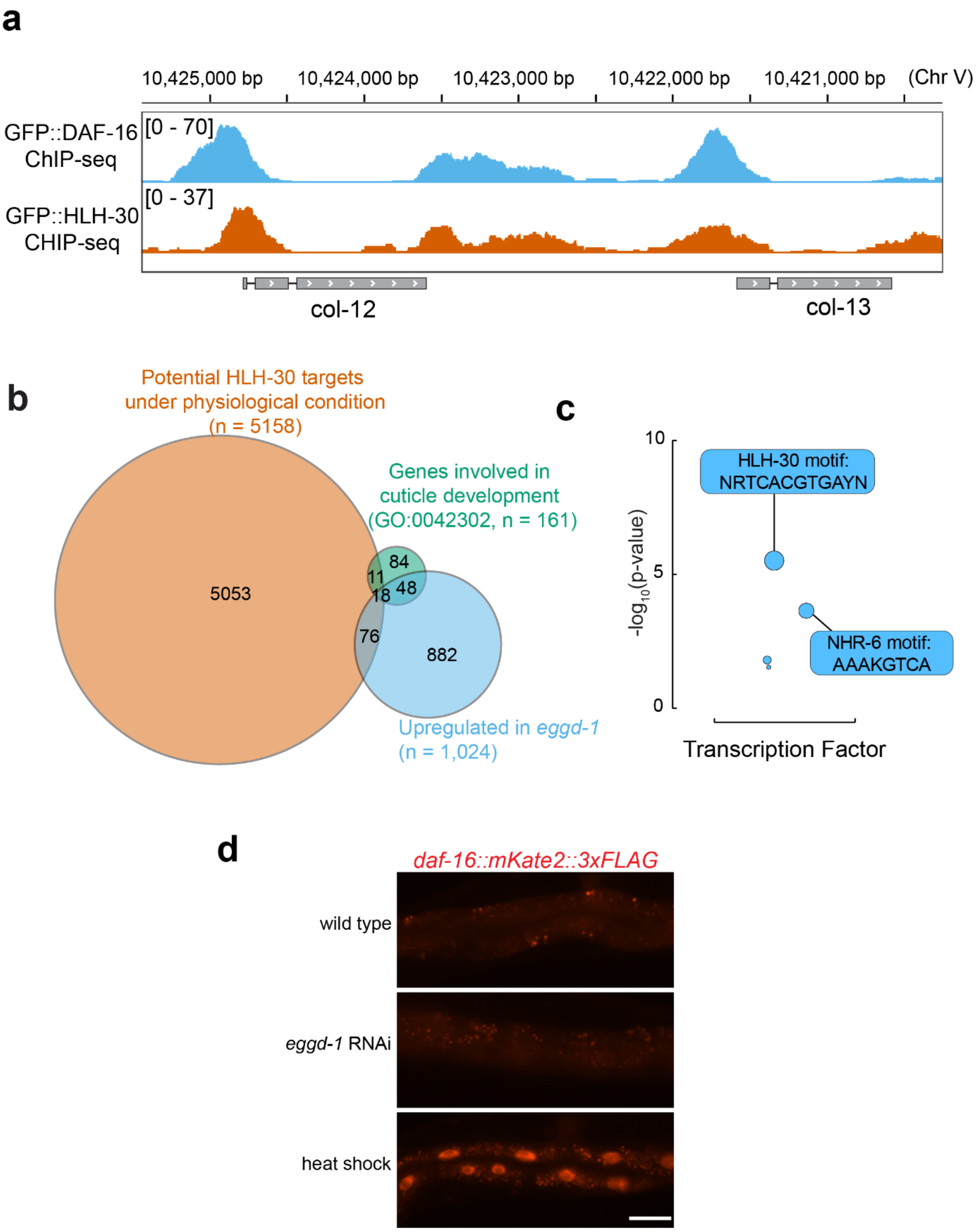

Supplementary Fig. 6: Expression and binding sites of HLH-30 and DAF-16.
a. Genome browser view of DAF-16 and HLH-30 CHIP data (modENCODE project) showing both transcription factors bind to promoter regions of *col-12* and *col-13*.

b. Venn diagram showing the overlap between HLH-30 target genes (n = 5158), upregulated genes in *eggd-1* mutants (n = 1024), and genes involved in cuticle development (GO term: 0042302, n= 161).

c. Transcription factor binding site enrichment analysis[80] showing overrepresentation of the HLH-30 and NHR-6 binding motifs at upregulated genes when *eggd-1* is mutated.

d. Widefield images of *DAF-16::mKate2* in adult hermaphrodites plated on OP50 or *eggd-1* RNAi food at 20°C. Heat-shocked worms were plated on a preheated plate with OP50 at 34°C for 1 hour. n = 7 worms imaged over one experiment. Scale bar = 20 µm.
