# Supplementary material for: C. elegans germ granules sculpt both germline and somatic RNAome
Source: Nat Commun. 2023 Sep 25;14:5965. doi: 10.1038/s41467-023-41556-4 (PMC10520050; doi:10.1038/s41467-023-41556-4)
Supplement: Supplementary file 3 — Description of Additional Supplementary Files [file 41467_2023_41556_MOESM3_ESM.pdf]

Description of Additional Supplementary Files

File name: Supplementary Data 1

Description: List of strains used in this study.
