# Supplementary material for: C. elegans germ granules sculpt both germline and somatic RNAome
Source: Nat Commun. 2023 Sep 25;14:5965. doi: 10.1038/s41467-023-41556-4 (PMC10520050; doi:10.1038/s41467-023-41556-4)
Supplement: Supplementary file 5 — Reporting Summary [file 41467_2023_41556_MOESM5_ESM.pdf]

nature portfolio

Corresponding author(s): Wen Tang
Last updated by author(s): 2023/08/30

Reporting Summary

Nature Portfolio wishes to improve the reproducibility of the work that we publish. This form provides structure for consistency and transparency in reporting. For further information on Nature Portfolio policies, see our Editorial Policies and the Editorial Policy Checklist.

Statistics

For all statistical analyses, confirm that the following items are present in the figure legend, table legend, main text, or Methods section.

n/a	Confirmed	
☐	☒	The exact sample size (*n*) for each experimental group/condition, given as a discrete number and unit of measurement
☐	☒	A statement on whether measurements were taken from distinct samples or whether the same sample was measured repeatedly
☐	☒	The statistical test(s) used AND whether they are one- or two-sided<br>*Only common tests should be described solely by name; describe more complex techniques in the Methods section.*
☐	☒	A description of all covariates tested
☐	☒	A description of any assumptions or corrections, such as tests of normality and adjustment for multiple comparisons
☐	☒	A full description of the statistical parameters including central tendency (e.g. means) or other basic estimates (e.g. regression coefficient) AND variation (e.g. standard deviation) or associated estimates of uncertainty (e.g. confidence intervals)
☐	☒	For null hypothesis testing, the test statistic (e.g. *F*, *t*, *r*) with confidence intervals, effect sizes, degrees of freedom and *P* value noted<br>*Give P values as exact values whenever suitable.*
☒	☐	For Bayesian analysis, information on the choice of priors and Markov chain Monte Carlo settings
☒	☐	For hierarchical and complex designs, identification of the appropriate level for tests and full reporting of outcomes
☐	☒	Estimates of effect sizes (e.g. Cohen's *d*, Pearson's *r*), indicating how they were calculated

Our web collection on statistics for biologists contains articles on many of the points above.

Software and code

Policy information about availability of computer code

Data collection	MetaMorph version 7.10.4.452, NIS-Elements 5.41.02, RNA-seq libraries were sequenced on Illumina platforms. Base calls were made using standard Illumina software.
Data analysis	ImageJ 1.54a, R-4.3.1, Data were analyzed using custom code freely available on github. Code written specifically for this can be found at github.com/benpastore/eggd_RNA_2023. Pipelines used to analyze mRNA and smRNA sequencing can be found at github.com/benpastore/nextflow_pipelines and github.com/benpastore/nextflow_smRNA, respectively. Any other code not listed here is available upon request.

For manuscripts utilizing custom algorithms or software that are central to the research but not yet described in published literature, software must be made available to editors and reviewers. We strongly encourage code deposition in a community repository (e.g. GitHub). See the Nature Portfolio guidelines for submitting code & software for further information.

Data

Policy information about availability of data

All manuscripts must include a data availability statement. This statement should provide the following information, where applicable:

- Accession codes, unique identifiers, or web links for publicly available datasets
- A description of any restrictions on data availability
- For clinical datasets or third party data, please ensure that the statement adheres to our policy

mRNA and small RNA sequencing data are deposited to NCBI GEO under GSE228858 and GSE228857 respectively. Additionally, both mRNA and smRNA sequencing

can be accessed using the GEO SuperSeries ascension number GSE228859. All Scripts used in data analysis are available at Github: https://github.com/benpastore/eggd_RNA_2023.

Research involving human participants, their data, or biological material

Policy information about studies with human participants or human data. See also policy information about sex, gender (identity/presentation), and sexual orientation and race, ethnicity and racism.

Reporting on sex and gender	n/a
Reporting on race, ethnicity, or other socially relevant groupings	n/a
Population characteristics	n/a
Recruitment	n/a
Ethics oversight	n/a

Note that full information on the approval of the study protocol must also be provided in the manuscript.

Field-specific reporting

Please select the one below that is the best fit for your research. If you are not sure, read the appropriate sections before making your selection.

☒ Life sciences ☐ Behavioural & social sciences ☐ Ecological, evolutionary & environmental sciences

For a reference copy of the document with all sections, see nature.com/documents/nr-reporting-summary-flat.pdf

Life sciences study design

All studies must disclose on these points even when the disclosure is negative.

Sample size	Sample sizes are indicated in figure legends or methods. No explicit power analysis was used. For live confocal/widefield fluorescence imaging experiments of C. elegans, sample sizes were determined by referring to the literature where similar experiments have been performed by other labs in the field: Marnik et al. 2019 (https://doi.org/10.1534/genetics.119.302670); Wan et al. 2021 (https://doi.org/10.15252/embj.2020105612); Placentino et al. 2021 (https://doi.org/10.15252/embj.2020105280); Schreier et al. 2022 (https://doi.org/10.1038/s41556-021-00827-2); Ouyang et al. 2022 (https://doi.org/10.1038/s41556-022-00940-w); and Thomas et al. 2023 (https://doi.org/10.15252/embj.2022112987) report imaging between 4 and 12 worms per experimental condition (biological replicates) for representative images and quantification of phenomena in the adult C. elegans germ line, which we adhered to for our experiments. For figure 4a/b CSR-1 piRNA (n = 20-30 worms per condition) sensor experiment we referred to Shirayama et al. 2012 (https://doi.org/10.1016/j.cell.2012.06.015).For Figure 4c, germ line RNAi competence, our sample size (3 independent biological replicates of >>100 embryos) is in excess of what is strictly necessary to show complete penetrance of embryonic lethality in eggd-1/2 mutants under pos-1 RNAi since we observed no experimental variation. For Figure 4d GFP RNAi inheritance (~50 worms per generation) sample size determination we referred to Spracklin et al. 2017 (https://doi.org/10.1534/genetics.116.198812). For figure 5 f/g we referred to Cipriani et al. 2021 (https://doi.org/10.7554/eLife.60833) to approximate the sample size necessary to quantify a moderate-to-low-penetrance germ line masculinzation phenotype.
Data exclusions	no data were excluded from analysis
Replication	figure 1 microscopy experiments were performed twice with similar results. figure 3 confocal microscopy experiments were performed once and agree with results from separate widefield microscopy experiments. The piRNA sensor experiment in figure 4a,b was performed once. Results from RNAi experiments in figure 4 c,d are composites of 3 separate experiments. figure 5 f/g are composites of two separate experiments with similar results. RNAi and microscopy experiments in figure 6 were repeated twice with similar results. RNAi and microscopy experiments in figure 7a were performed once, and figure 7b were performed twice with similar results. RNA sequencing experiments were performed using biological duplicates of ~100,000 worms each, processed in parallel for both mRNA sequencing and sRNA sequencing. Biological duplicate samples correlate well and cluster together in a principle component analysis.
Randomization	No random allocation was performed. Samples were allocated to experimental groups by genotype or by RNAi condition. We controlled for covariates by maintaining consistent growth conditions (temperature, growth medium).
Blinding	No blinding was done during experimental data collection and analysis. Blinding was not relevant because effects of mutants tested are generally categorical with clear binary outcomes (e.g. embryonic viability, signal on vs. off) or determined using systematic quantification (RNA-seq).

Reporting for specific materials, systems and methods

We require information from authors about some types of materials, experimental systems and methods used in many studies. Here, indicate whether each material, system or method listed is relevant to your study. If you are not sure if a list item applies to your research, read the appropriate section before selecting a response.

Materials & experimental systems

n/a	Involved in the study
☒	☐ Antibodies
☒	☐ Eukaryotic cell lines
☒	☐ Palaeontology and archaeology
☐	☒ Animals and other organisms
☒	☐ Clinical data
☒	☐ Dual use research of concern
☒	☐ Plants

Methods

n/a	Involved in the study
☒	☐ ChIP-seq
☒	☐ Flow cytometry
☒	☐ MRI-based neuroimaging

Animals and other research organisms

Policy information about studies involving animals; ARRIVE guidelines recommended for reporting animal research, and Sex and Gender in Research

Laboratory animals	C. elegans
Wild animals	the study did not use wild animals
Reporting on sex	we used hermaphrodite worms for our reported analyses. We did not perform sex-based analysis because C. elegans does not naturally produce true females, and males are a rare subset of C. elegans populations (~0.1%), so hermaphrodites are the most relevant sex to investigate for the purpose of this report.
Field-collected samples	no field-collected samples were used in this study
Ethics oversight	no ethical guidance is required for work with C. elegans

Note that full information on the approval of the study protocol must also be provided in the manuscript.
